# Posterior decompression and short segmental pedicle screw fixation combined with vertebroplasty for Kümmell’s disease with neurological deficits

**DOI:** 10.3892/etm.2012.833

**Published:** 2012-11-26

**Authors:** GUANG-QUAN ZHANG, YAN-ZHENG GAO, JIA ZHENG, JIAN-PING LUO, CHAO TANG, SHU-LIAN CHEN, HONG-QIANG WANG, KE LIU, RUI-GANG XIE

**Affiliations:** 1 Departments of Orthopedics, Henan Province People’s Hospital, Zhengzhou, Henan 450003, P.R. China; 2 Radiology, Henan Province People’s Hospital, Zhengzhou, Henan 450003, P.R. China

**Keywords:** Kümmell’s disease, vertebroplasty, decompression operation, intravertebral vacuum phenomenon

## Abstract

The aim of this study was to investigate the treatment of Kümmell’s disease with neurological deficits and to determine whether intravertebral clefts are a pathognomonic sign of Kümmell’s disease. A total of 17 patients who had initially been diagnosed with Kümmell’s disease were admitted, one patient was excluded from this study. Posterior decompression and vertebroplasty for the affected vertebrae were conducted. Pedicle screw fixation and posterolateral bone grafts were performed one level above and one level below the affected vertebrae. Vertebral tissue was extracted for histopathological examination. The mean time of follow-up was 22 months (range, 18 to 42 months). The anterior and middle vertebral heights were measured on standing lateral radiographs prior to surgery, one day postoperatively and at final follow-up. The Cobb angle, the visual analog scale (VAS) and the Frankel classification were used to evaluate the effects of the surgery. The VAS, anterior and middle vertebral heights and the Cobb angle were improved significantly one day postoperatively and at the final follow-up compared with the preoperative examinations (P<0.05). No significant differences were observed between the one-day postoperative results and those at final follow-up (P>0.05). The neurological function of all patients was improved by at least one Frankel grade. All patients in this study exhibited intravertebral clefts, and postoperative pathology revealed bone necrosis. One patient (not included in this study) showed an intravertebral cleft, but the pathology report indicated a non-Hodgkin’s lymphoma. The intravertebral cleft sign is not pathognomonic of Kümmell’s disease. Posterior decompression with short-segment fixation and fusion combined with vertebroplasty is an effective treatment for Kümmell’s disease with neurological deficits.

## Introduction

Delayed post-traumatic vertebral collapse, characterized by painful kyphosis that develops several weeks or months following an injury after a symptom-free period, was first publicly presented by the German surgeon Hermann Kümmell in 1895 (1). The development of the disease has three phases. In the first phase, patients initially experience back pain, which subsides and leads to an asymptomatic period. In the second phase, the pain recurs weeks to months after the initial incident without further apparent trauma. In the third phase, patients develop progressive angular kyphosis. With the advent of radiography, the progressive angular kyphosis was attributed to a delayed post-traumatic vertebral compression fracture. The outstanding radiological findings of Kümmell’s disease consist of an intravertebral cleft (either intravertebral vacuum cleft or fluid collection) combined with a collapsed vertebra. More recently, multiple synonymous terms have been used to describe Kümmell’s disease, including delayed post-traumatic vertebral collapse (2,3), vertebral osteonecrosis (4,5), intravertebral pseudarthrosis (4,6), fracture non-union (6) and intravertebral cleft (7). However, whether intravertebral clefts are a pathognomonic sign of Kümmell’s disease is controversial. Certain studies have demonstrated that intravertebral clefts are a benign sign, whereas others have reported that intravertebral clefts occur rarely in patients with spinal infection and in patients with multiple myeloma (8,9).

For Kümmell’s disease with persistent pain and without neurological symptoms, percutaneous vertebroplasty (PVP) (4,10) or kyphoplasty (PKP) (5,6,11) achieves good results. For patients with neurological deficits, PVP and PKP are unsuitable. In the past, anterior decompression with bone grafting fusion (12), posterior decompression with pedicle subtraction osteotomy (PSO) (2,3,13,14) or a combined anterior and posterior approach operation (14) were used, but these procedures have long surgery times and cause increased hemorrhage and multiple complications. In the current study, posterior decompression and vertebroplasty were used to treat the affected vertebrae. Pedicle screw fixation and posterolateral bone grafts were performed at one level above and one level below the affected vertebrae for Kümmell’s disease with neurological deficits and improved results were achieved. Through a review of the literature (15–18) combined with our own study, we intend to further investigate whether intravertebral clefts are a pathognomonic sign of Kümmell’s disease and to determine a suitable treatment method for Kümmell’s disease with neurological deficits.

## Materials and methods

### Patients

The cohort consisted of 16 patients, 7 males and 9 females, 69–82 years old (average, 74 years), admitted to Henan Province People’s Hospital between October 2007 and August 2010. Ten of the patients had a history of minor trauma. The duration of their symptoms ranged from 2 to 8 months. Two patients were undergoing long-term hormone replacement therapy. Eight patients had hypertension and four cases had diabetes mellitus. The mean bone mineral density was −3.65 standard deviation (SD). The treated vertebrae were as follows: T7 (n=1), T9 (n=1), T10 (n=2), T11 (n=3), T12 (n=6), L1 (n=2) and L3 (n=1). Preoperative imaging included standing anteroposterior and lateral radiographs, CT scans and MRI examination. Five patients underwent stress views (standing extension and flexion lateral radiographs) in addition to the standard radiographs. Postoperative standing radiographs were taken to assess the effect of the surgery. This study was conducted in accordance with the Declaration of Helsinki and with approval from the Ethics Committee of the Henan Province People’s Hospital. Written informed consent was obtained from all participants.

### Surgical procedure

The patients were operated on under general anesthesia and placed in the prone position. Pillows were used to support the upper chest and pelvis and the operating table was adjusted to enable maximum extension of the spinal column. This postural reduction generally restored most of the body height of the fractured vertebrae. Using a standard posterior midline approach, pedicle screws were placed promptly into the vertebrae one level above and below the affected vertebra through a distraction rod to restore the vertebral body height further. The diseased vertebral laminae and ligamentum flavum were resected to decompress the spinal cord. A puncture needle was driven into the affected vertebral body to establish a working channel. A biopsy needle was used to collect a specimen. Under fluoroscopic guidance, bone cement was injected into the vertebral body. Intraoperative exploration revealed no compression of the dural sac and no leakage of bone cement in the spinal canal. Posterolateral fusion with autogenous bone grafts from the decompression laminectomy was performed.

### Evaluation

Vertebral height was measured in millimeters along the vertebral borders at the anterior and middle of the vertebral body. The Cobb angle was measured as the angle between the upper endplate of the upper vertebra of the fractured vertebra and the lower endplate of the lower vertebra of the fractured vertebra. The visual analog scale (VAS), which ranges from 0 (no pain) to 10 (maximal pain), was used to assess pain severity. Frankel classification was used to assess neurological status and the development of surgical complications was observed.

### Statistical analysis

SPSS 17.0 statistical software (SPSS, Inc., Chicago, IL, USA) was used for analysis. The data are presented as the mean ± standard deviation. One-way ANOVA was used to evaluate the changes in the VAS, Cobb angles and vertebral body heights based on the data obtained preoperatively, one day postoperatively and at final follow-up. A multiple comparison was conducted using the least significant difference test. P<0.05 was considered to indicate a statistically significant difference.

## Results

**Figure 1.**
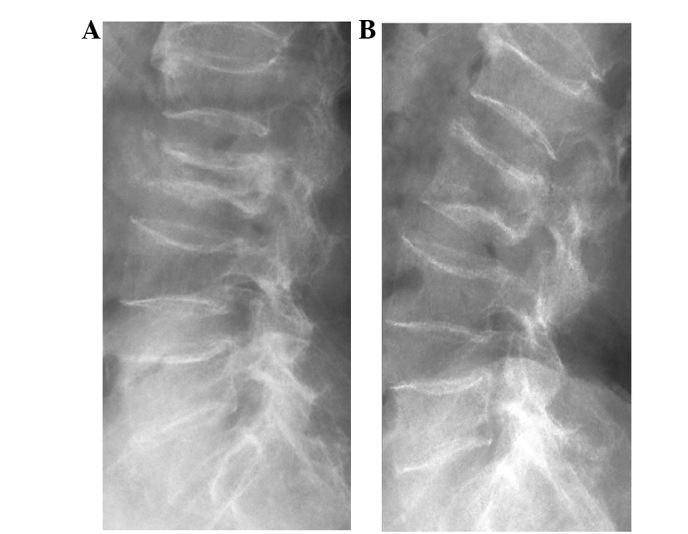
X-ray views of an 80-year-old female patient suffering from Kümmell’s disease at L3. (A) Standing flexion lateral X-ray view showed L3 vertebra compression. (B) Standing extension lateral X-ray view showed L3 vertebra height increased.

Preoperative standing lateral radiographs and intraoperative prone position lateral radiographs, as well as preoperative extension and flexion radiographs of five patients, were compared and it was identified that vertebral height varied with postural changes. Stress view radiographs showed that vertebral height decreased with flexion and increased with extension (Fig. 1).

**Figure 2.**
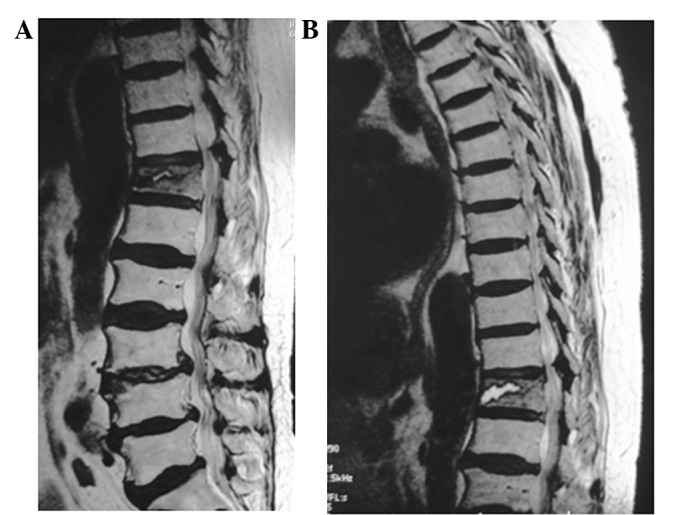
MRI scans for a 70-year-old female patient suffering from Kümmell’s disease at T12. (A) The lumbar MRI, T2-weighted image showed a mixed signal of gas and liquid at T12 and (B) 8 min later, the thoracic MRI, T2-weighted image showed a hyperintense signal of liquid at T12.

All 16 patients presented with intravertebral cleft signs during the preoperative examination. The following radiological patterns were identified as signs of an intravertebral cleft: i) a gas-filled transverse band in the vertebral body on a conventional radiograph (5 cases); ii) a gas-filled transverse band in the vertebral body on a CT image (9 cases, 3 of which exhibited adjacent intradiscal gas at the same time) and iii) a gas or fluid signal on an MRI scan (preoperative MRI scans of all 16 patients). In one case, a lumbar MRI T2-weighted image revealed mixed signals of gas and liquid at T12. After 8 min, the thoracic MRI T2-weighted image displayed an apparent inconsistency and a hyperintense fluid signal at T12 (Fig. 2).

**Figure 3.**
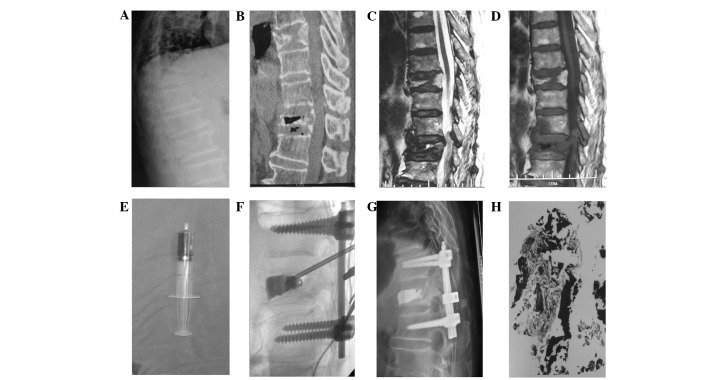
A 76-year-old male patient with bilateral lower limb numbness and weakness for half a year prior to admission and a preoperative BMD of −3.8 standard deviation (SD). (A) Preoperative standing lateral radiograph revealed T12 compression fracture and kyphosis. (B) Preoperative CT showed the intravertebral vacuum sign and gas in the adjacent disc. (C and D) Preoperative MRI displayed a mixed signal of liquid and gas within the vertebral body. (E) Liquid collected intraoperatively from the vertebral body. (F) Intraoperatively, the vertebral height was restored and the bone cement filled the cleft well with no leakage. (G) Standing lateral radiographs showed no decrease in vertebral height and no kyphosis recurrence after a follow-up of 18 months. (H) Postoperative pathology revealed necrosis of the bone. BMD, bone mineral density.

**Figure 4.**
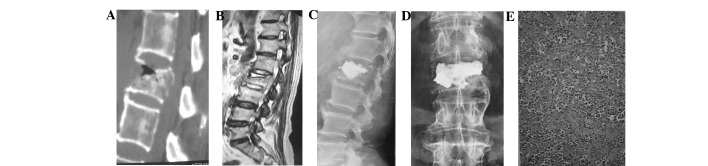
Imaging and pathology results of a 72-year-old male patient. (A) CT displayed the intravertebral vacuum sign at L2. (B) MRI displayed a fluid signal at L2. (C and D) Anteroposterior and lateral X-ray films after surgery showed that the bone cement had filled the cleft well. (E) Postoperative pathology revealed non-Hodgkin’s lymphoma (H&E staining).

The mean surgery time was 110 min (range, 90–140 min), and the mean estimated blood loss was 250 ml (range, 150–500 ml). The mean volume of polymethylmethacrylate (PMMA) was 7.2 ml (range, 4.5–12 ml). A spinal dural tear occurred in one case. Intraoperative biopsies from all 16 cases reported bone necrosis (Fig. 3). Clinically, one patient was identified who had no neurological deficits (and so was excluded from the group), whose CT displayed the vacuum phenomenon (Fig. 4A) and whose MRI scan displayed a liquid signal (Fig. 4B); the pathology report revealed non-Hodgkin’s lymphoma (Fig. 4E).

The patients underwent follow-up after 18–42 months (mean, 22 months). The mean VAS score, the anterior and middle height of the affected vertebrae and the Cobb angle improved significantly from prior to the surgery to one day postoperatively (P<0.01). The improvement was maintained from one day postoperatively to the final follow-up (P>0.05; Table I). No patient received a grade A under the Frankel classification. Preoperatively, two patients were classified as grade B, five were grade C and nine were grade D. One day postoperatively, one patient was grade B, three were grade C, seven were grade D and five were grade E. At final follow-up, two patients were grade C, five were grade D and nine were grade E. The neurological function of each patient was improved by at least one level at the final follow-up (Table II). One patient developed a superficial skin infection. No obvious loosening of internal fixation, breakage or bone cement displacement occurred.

## Discussion

Maldague *et al*(19) first reported the intravertebral vacuum cleft sign, and the authors considered gas accumulation (vacuum cleft sign) in the vertebral body on plain X-rays as pathognomonic of Kümmell’s disease. The vacuum phenomenon is more evident in the extended position and may reduce or disappear in the flexed position. The gas noted on the plain radiographs was expected to be hypointense on both the MRI T1 and T2 sequences. However, the majority of authors have reported either a homogeneous fluid or gas signal on the MRI sequences of patients with the intravertebral vacuum phenomenon. Malghem *et al*(8) plausibly explained this phenomenon. Patients with the vacuum sign were serially imaged, and the MRI demonstrated that the initially gas-filled cleft appeared hypointense. However, following prolonged supine positioning, a hyperintense signal appeared on the T2 sequences, indicating the presence of fluid instead of gas. We also observed this phenomenon in one patient. The lumbar MRI T2-weighted image showed a mixed signal of gas and liquid at T12. After 8 min, the thoracic MRI T2-weighted image showed a hyperintense liquid signal at T12, which suggests that the contents (fluid and gas within the vertebral body) are variable over time.

Whether intravertebral clefts are a pathognomonic sign of Kümmell’s disease is controversial. Certain studies have demonstrated that intravertebral clefts are a benign sign, whereas others have reported that intravertebral clefts occur rarely in patients with spinal infections and in patients with multiple myeloma (8,9). We identified a patient (excluded from the study) with a CT that displayed a vacuum phenomenon (Fig. 4A) and an MRI that displayed a liquid sign (Fig. 4B). The patient was diagnosed with Kümmell’s disease based on the clinical and radiological signs. The vacuum cleft was filled well with PMMA (Fig. 4C and D). However, the pathology report revealed non-Hodgkin’s lymphoma (Fig. 4E). To the best of our knowledge, no non-Hodgkin’s lymphoma with vacuum cleft has been reported. Therefore, intravertebral clefts are not pathognomonic of Kümmell’s disease, but they are highly suggestive of the disease. Thus, we consider that it is necessary to confirm Kümmell’s disease with bone necrosis under biopsy.

The pathogenesis of the vertebral vacuum phenomenon remains controversial and it has been mainly theorized to involve vertebral avascular necrosis (4,19), vertebral fracture nonunion or pseudarthrosis (6) or intradiscal gas leakage through the endplate fractured into the vertebral body (20). In the current study, only two patients had factors that predispose to bone necrosis (long-term corticosteroid application history). The remaining patients had no other predisposing factors. The theory of vertebral avascular necrosis alone does not explain the pathogenesis of the disease. In the current study, nine patients exhibited a gas signal in the affected vertebral body based on CT but only three cases had gas in the adjacent disk. Therefore, the theory that intravertebral gas originates from the adjacent disk alone does not explain the intravertebral vacuum phenomenon. In addition, we compared the preoperative standing lateral radiographs and intraoperative prone lateral radiographs, as well as the preoperative extension and flexion radiographs, of five patients. We found that vertebral height varied with postural changes, in accordance with the report by Yang *et al*(6). These findings support the theory of vertebral fracture nonunion or pseudarthrosis. Thus, we advocate the complete filling of the cleft with cement to maximize stabilization of the pseudarthrosis. In the current study, the mean amount of cement injected was 7.2 ml. According to the literature, as well as our imaging results and clinical data, the pathogenesis of the vertebral cleft phenomenon requires a combination of avascular bone necrosis, fracture non-healing and adjacent intradiscal gas diffusion.

The treatment strategies for Kümmell’s disease differ between patients with neurological symptoms and those without neurological symptoms. For patients without neurological symptoms, the objective is to eliminate motion at the fracture site and restore the spinal curvature. Certain authors have reported that PVP (4,8) or PKP (5,6,9) achieves good clinical results for Kümmell’s disease without neurological symptoms. For neurologically impaired patients, the aim of surgery is to decompress the spinal cord, restore the spinal physiological curvature and maintain spinal stability. The surgical modes include anterior, posterior or combined anterior and posterior approaches. Anterior decompression and fusion with intervertebral tricortical graft or ceramic glass spacers has favorable results. These procedures are the most efficient for decompressing the spinal cord since the locus of pathology (deficient anterior and middle spinal columns) is directly addressed, and they provide anterior column support. Anterior approach surgery has a high fusion rate (95.5–100%) and the postoperative kyphosis correction angle has a mean of 10.4–18°. At final follow-up, the corrected degree decreased by 4.8–8°. The drawback of the anterior approach in pleural and extrapleural operations is that it may cause pulmonary complications in injuries of the thoracolumbar junction, where most cases of intravertebral vacuum occur, and it may affect gastrointestinal function in retroperitoneal surgery. Moreover, in the anterior approach, the stabilization of the spine may fail due to the osteoporotic bone. Surgeries that use the posterior approach include decompression and PSO (2,3,13,14). The fusion rate of the posterior approach operation is 62.5–100%, and the immediate postoperative kyphosis correction angle is 14.6–25.7°. The average loss of correction at final follow-up is 2.4–8.8°. PSO surgery often requires the fixation of the vertebral bodies above and below the affected vertebra; thus, adjacent vertebral disease often occurs. A combined anterior and posterior approach has a good fusion rate (100%), with a kyphosis angle correction of 11.2° postoperatively and a loss of 4.2° at final follow-up. However, the surgery time is longer (351 min) and the blood loss is higher (2892 ml) (14).

Patients with Kümmell’s disease with neurological symptoms are often older and have a variety of diseases; thus, the patients do not easily tolerate the aforementioned surgical methods. Therefore, the development of a minimally invasive and effective treatment is required. Surgeons have performed open posterior decompression and short-segment fixation for Kümmell’s disease with neurological symptoms, followed by vertebroplasty (15–18) or kyphoplasty (17) under direct visualization. This surgical method provides several advantages. Posterior decompression relieves nerve compression with short segment fixation and fusion reduces the fusion segment and the influence of long segmental spinal function. Vertebral bone cement provides anterior support to minimize posterior pedicle screw stress. Furthermore, bone cement leakage may be avoided under direct vision. Matsuyama *et al*(18) used calcium phosphate cement, which polymerizes at lower temperatures. The results included effective pain relief (from 8.6, preoperatively, to 2, postoperatively, on the VAS), nerve function and kyphosis restoration (vertebral height from 41% preoperatively to 74% postoperatively and 68% at final follow-up). In the current study, we used PMMA for vertebroplasty which achieved effective pain relief (the mean preoperative VAS score of 8.49 was reduced to 2.09 one day postoperatively and 2.29 at final follow-up) and good postoperative kyphosis correction (the anterior and central vertebral body height were enhanced by ∼1 cm and Cobb’s angle correction was 18.29° one day postoperatively). Follow-up examinations were conducted for ≥18 months. At the final follow-up, a slight reduction in the vertebral height and a kyphosis correction of 1.11° were observed compared with those at one day after the surgery. However, these differences were not statistically significant. The patients recovered neurologically, and nerve function improved by least one Frankel grade at final follow-up. The mean surgery time was 110 min (range, 90–140 min) and the mean estimated blood loss was 250 ml (range, 150–500 ml). Thus, posterior decompression with short-segment fixation and fusion combined with vertebroplasty is an effective treatment for Kümmell’s disease with neurological symptoms, especially for patients who are not able to tolerate long surgery times and massive blood loss. However, a previous study hypothesized that the osteolysis rate among patients with Kümmell’s disease is greater than the rate of bone callus formation. Following PVP or PKP, accelerated osteolysis occurs and may displace the bone cement (21). Two case reports have focused on bone cement displacement following PVP (22) or PKP (23) alone for Kümmell’s disease without neurological deficits. Therefore, greater numbers of patients and longer follow-up times are required to verify the efficiency of posterior decompression with short segmental pedicle screw fixation and fusion combined with vertebroplasty for Kümmell’s disease with neurological deficits.

## Figures and Tables

**Table I.** Evaluation indices prior to and following surgery.

	Cobb angle height (cm)	Anterior vertebral height (cm)	Middle vertebral height (cm)	VAS score
Preoperative	29.63±3.97	1.05±0.23	1.51±0.26	8.49±0.43
One day postoperatively	11.34±2.25^a^	2.40±0.27^a^	2.47±0.29^a^	2.09±0.36^a^
Final follow-up	12.45±2.35^a^,^b^	2.28±0.25^a^,^b^	2.42±0.34^a^,^b^	2.29±0.31^a^,^b^
F-value	191.70	141.735	55.668	1601.407
P-value	0.000	0.000	0.000	0.000

a P<0.05 vs. preoperative values;

b P>0.05 vs. one day postoperative values.

**Table II.** Patient numbers prior to and following surgery by Frankel classification.

	Frankel classification (patient number)				
Time point	A	B	C	D	E
Preoperative	0	2	5	9	0
One day postoperatively	0	1	3	7	5
Final follow-up	0	0	2	5	9
